# New Directions in Imaging Neuroendocrine Neoplasms

**DOI:** 10.1007/s11912-021-01139-2

**Published:** 2021-11-04

**Authors:** Julie Refardt, Johannes Hofland, Damian Wild, Emanuel Christ

**Affiliations:** 1 Department of Internal Medicine, Section of Endocrinology, ENETS Center of Excellence, Erasmus Medical Center, Rotterdam, the Netherlands; 2 ENETS Center of Excellence for Neuroendocrine and Endocrine Tumors, University Hospital Basel, Petersgraben 4, 4031 Basel, Switzerland; 3 Department of Endocrinology, Diabetology and Metabolism, University Hospital Basel, Petersgraben 4, 4031 Basel, Switzerland; 4 Division of Nuclear Medicine, University Hospital Basel, Petersgraben 4, 4031 Basel, Switzerland

**Keywords:** Neuroendocrine neoplasms, Peptide hormone receptors, Somatostatin receptor antagonist, GLP-1 receptor imaging, CCK2-receptor imaging

## Abstract

**Purpose of Review:**

Accurate imaging is crucial for correct diagnosis, staging, and therapy of neuroendocrine neoplasms (NENs). The search for the optimal imaging technique has triggered rapid development in the field. This review aims at giving an overview on contemporary imaging methods and providing an outlook on current progresses.

**Recent Findings:**

The discovery of molecular targets due to the overexpression of specific peptide hormone receptors on the NEN’s surface has triggered the development of multiple radionuclide imaging modalities. In addition to the established imaging technique of targeting somatostatin receptors, several alternative radioligands have been developed. Targeting the glucagon-like peptide-1 receptor by exendin-4 has a high sensitivity in localizing insulinomas. For dedifferentiated NENs, new molecular targets such as the C-X-C motif chemokine-receptor-4 have been evaluated. Other new targets involve the fibroblast activation protein and the cholecystokinin-2 receptors, where the ligand minigastrin opens new possibilities for the management of medullary thyroid carcinoma.

**Summary:**

Molecular imaging is an emerging field that improves the management of NENs.

## Introduction

Neuorendocrine neoplasms (NENs) are rare tumors with increasing prevalence deriving from neuroendocrine cells localized mainly in the intestine, pancreas and lung [1, 2]. In about half of the patients, the diagnosis is established at a non-resectable stage due to unspecific clinical syndromes and slow tumor growth with late symptomatic manifestation. However, incidental detection at an early stage of disease has been reported due to improved diagnostic procedures [3]. The classification of NENs depends on their origin and extension, with the grading being based on histological differentiation [4]. Well-differentiated NENs are classified as grade 1, 2, or 3 neuroendocrine tumors (NETs), based on the mitotic counts and Ki67 index, while poorly differentiated NENs are categorized as grade 3 neuroendocrine carcinomas (NEC) [5•]. A further distinction—in about 25% of patients—involves the ability of the tumors to secrete hormones leading to specific symptoms, separating them into functioning and non-functioning tumors [6]. Treatment is as diverse as these tumors, dependent on stage, grade, and clinical presentation and involves surgery, imaging-guided local ablative or vascular therapy, radionuclide therapy, targeted treatment, biotherapy, and chemotherapy.

Accurate and informative imaging is crucial for correct diagnosis, staging, and treatment decision. However, a standardized approach is difficult due to the diversity of primary tumor sites and metastases. Accordingly, the search for the optimal imaging technique has triggered rapid development and improvement of this field. One of the most revolutionary improvements was the discovery of molecular imaging targets due to the overexpression of specific peptide hormone receptors on the cell surface of NENs [7].

**Fig. 1:**
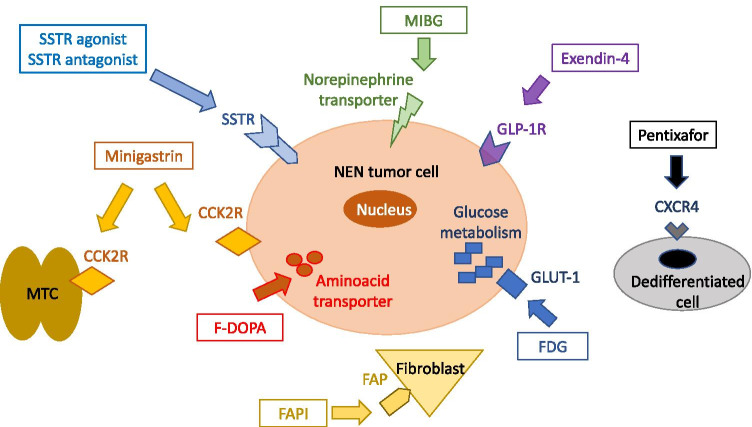
Overview of currently used molecular targets for imaging of NENs. CCK2-R = cholecystokinin-2 receptor, CXCR4 = C-X-C motif chemokine-receptor-4, FAP = fibroblast activation protein, FDG = fluorodeoxyglucose, F-DOPA = fluoro-dopa, GLP1R = GLP-1-receptor, MIBG = metaiodobenzygluanidine, NEN = neuroendocrine neoplasm, SSTR = somatostatin receptor

This review aims at giving an overview on the most commonly used imaging modalities to diagnose and stage NENs today, but also providing an outlook into new developments and future techniques. Figure 1 summarizes the molecular targets currently used.

## Conventional Morphological Imaging

### Computed Tomography

Computed tomography (CT) is the main modality used for the evaluation of NENs due to its wide availability, speed, and low cost [8•]. Multidetector CT scanners, generating hundreds of thin transversal images, allow for detailed evaluation through reconstructed images. Through the administration of intravenous (i.v.) contrast, multiphasic or dynamic imaging is performed, which is essential for the evaluation of various structures.

The importance of having a contrast-enhanced image is best shown for the assessment of the liver, which includes non-contrast images, arterial phase for the imaging of hepatic and mesenteric arteries, portal venous phase for the visualization of hepatic and mesenteric veins as well as the hepatic parenchyma and lastly delayed phase for additional characterization of possible liver lesions. However, the standardized evaluation of liver lesions can be difficult. While many NEN lesions are hypervascular and therefore best seen on arterial phase images, an analysis of NEN liver metastasis revealed 16% hypovascular lesions, which are best seen in the delayed phase images [9]. Multiphasic CT is also important for the evaluation of treatment response. Here, treatment-specific effects should be taken into consideration, since some can influence the enhancement patterns of NEN liver metastases as seen with the mTOR inhibitor everolimus [10].

When CT is used together with functional imaging as positron emission tomography (PET), often only a low-dose CT is performed in order to enable anatomical correlation of the PET findings. However, since small (< 5 mm) lesions may be missed by PET, i.v. contrast-enhanced CT of liver and pancreas in late arterial phase and whole body in venous phase are preferred in these indications.

CT shows a high-detection sensitivity for the majority of NENs and is the recommended morphological imaging technique [8•, 11]. However, gastric, duodenal, colonic, and rectal NET are often diagnosed by endoscopy or endoscopic ultrasound. Accordingly, CT imaging in gastric NEN is only required for large (> 2 cm) or invasive tumors. Diagnosis of duodenal NENs can be challenging and distension of the duodenum with water as well as imaging during i.v. contrast enhancement is recommended to localize these small contrast-enhancing tumors.

For the evaluation of small intestinal lesions, CT-enterography can be considered, which helps detecting those usually contrast-enhancing small tumors. Often diagnosed at a late stage, small intestinal NENs can present with mesenteric metastases. These show as an irregular soft tissue mass on CT, including sometimes radiating fibrotic strands and calcifications [12]. The description of the involved arterial vessels is crucial if a surgical intervention is considered.

Pancreatic NENs are typically hypervascular masses with enhancement in the arterial phase [8•]. Contrast enhancement patterns were described to correlate with tumor grade, with higher-grade tumors showing venous and delayed phase enhancement [13]. Additional signs for more aggressive tumors are ill-defined margins, vascular invasion, tumor size, and pancreatic duct dilation [14]. Insulin-secreting pancreatic NENs (insulinomas) are typically small (0.5–2 cm) and hypervascular on early and late postcontrast images, while non-functional pancreatic NENs tend to be larger, can be cystic, or show necrotic changes [8•]. Liver metastases from insulinomas can present with perilesional steatosis on CT.

CT has only a minor role in the diagnosis of colonic or rectal NENs, which are better staged by magnetic resonance imaging (MRI) or endoscopic ultrasound, but its use is recommended for the evaluation of distant metastases.

For thoracic NENs, i.v. contrast-enhanced chest CT is the morphological imaging technique of choice [15]. Thin section CT chest scanning is useful to establish localization and metastatic spread [16].

### Magnetic Resonance Imaging

For soft tissue characterization, MRI has a superior detection rate than CT [17]. However, this difference is often not considered clinically relevant due to the longer examination time and availability of MRI. The better tissue contrast is particularly important for the evaluation of the liver, pancreas, bone, and brain [18, 19]. A recent comparison of pancreatic lesions of 73 multiple endocrine neoplasia type 1 (MEN1) patients with MRI, CT, and endoscopic ultrasound, showed MRI to be the most sensitive imaging technique (89% vs 86% vs 75%) according to agreement and reliability measures [20]. For liver imaging, MRI has the additional advantage of an available hepatocyte-specific contrast medium. Furthermore, it does not expose patients to radiation, which makes it an especially attractive imaging tool for regular surveillance in younger patients.

MRI protocols should include T1-(T1W) and T2-weighted (T2W) MR sequences, dynamic three-dimensional (3D) sequence before and after intravenous administration of a gadolinium chelate with multi-arterial, venous, and delayed acquisition and diffusion-weighted (DWI) sequences [21]. A typical NEN appears as a dark lesion in T1W and bright lesions in T2W images. Otherwise, contrast enhancement and morphological characteristics are often similar to CT; however, MRI is more likely to detect small lesions due to its better soft tissue contrast. MRI also has superior diagnostic accuracy and sensitivity compared to CT in enterography [22–24].

DWI is a valuable tool to enhance lesion-to-background contrast. In a recent retrospective analysis on 45 pancreatic NENs [25], DWI, and T2W sequences were the most accurate to detect the lesions. Confirmation of this data would enable a shorter test protocol without contrast medium administration. MRI also plays a role perioperatively, where MR cholangiopancreatography can be helpful to evaluate the pancreatic duct and biliary system.

## Molecular Imaging

### Somatostatin Receptor Imaging

Somatostatin receptors (SSTRs) are expressed on the surface of the majority of well-differentiated NENs with the expression density decreasing in poorly differentiated NENs [26]. Evaluating the SSTR expression in a NEN patient improves tumor localization and staging, but also opens new treatment options with peptide receptor radionuclide therapy (PRRT) in patients with sufficient expression [27]. Patients with no or insufficient SSTR expression on the other hand have been shown to have inferior prognosis with shorter survival time [28, 29]. While the SSTR subtype 2 has been the sole focus for imaging and therapy for a long time, the subtypes 3 and 5 have recently gained importance.

#### Somatostatin Receptor Scintigraphy

^111^Indium-pentetreotide (Octreoscan) was the first commercially approved technique for the diagnosis and staging of SSTR-expressing tumors and the gold standard technique for many years [27]. ^111^Indium (In) is a γ-emitting isotope, which is linked to the somatostatin analog (SSA) octreotide using the chelator DTPA (Diethylene-triaminepentaacetic acid) resulting in ^111^In-DTPA-octreotide or better known as ^111^In-pentetreotide. This radiotracer binds preferably to SSTR subtypes 2 and 5 on the cell surface of NENs and can be detected with a gamma camera [27]. The anatomical localization was improved after the development of single-photon emission computed tomography (SPECT), and more so with SPECT/CT fusion technique [30].

A downside of the Octreoscan is its—compared to current techniques—poorer resolution, high-radiation dose and lengthy process for injection and scanning. Having a half-life of 2.8 days, images are optimally obtained 4 h and 24 h after injection which is cumbersome for the patients [31]. ^111^In-pentetreotide is cleared almost entirely over the kidneys and patient radiation dose is around 12 mSV [27].

Overall, it has a lower tumor-to-normal-tissue contrast and also a poorer sensitivity for liver metastases and multifocal primary [32–34] and low detection rate for medullary thyroid cancer and insulinoma, since those show variable expression of SSTR2 [35].

#### ^68^Gallium-SSTR Imaging

Due to its greater spatial resolution, lower radiation dose and improved diagnostic accuracy as well as patients convenience, ^68^Gallium-SSTR-PET has replaced SSTR scintigraphy as the standard imaging technique for NEN staging [8•].

Using the chelator DOTA (tetra-zacyclododecane-tetra-acetic acid), the radiometal ^68^Gallium (Ga) is conjugated with an SSA peptide. While ^68^Ga-DOTATOC and -NOC use octreotide (Tyr-3/1-Nal3) with affinity for the SSTR subtypes 2, 3, and 5, ^68^Ga -DOTATATE uses octreotate which has an enhanced affinity for the SSTR subtype 2 [36]. Clinically, there is no real difference between ^68^Ga-DOTATOC- and ^68^Ga-DOTATATE-based PET imaging [37, 38], their respective use often depends on the availability of the centers. The dosage for PET imaging is 2 MBq of ^68^Ga-DOTA-SSA per kg body weight (up to 200 MBq) i.v. and a PET-CT is run 60 min after injection. Good hydration of patients before the application is recommended. ^68^Ga-DOTA-SSA is excreted through the kidneys. Patient radiation dosage is around 2.9 mSv [8•], thereby approximately 25% of the radiation burden of ^111^In-pentetreotide.

^68^Ga-SSTR-PET/CT has a high sensitivity of 88–93% and specificity of 88–95% [39, 40] for most well-differentiated NENs. Sensitivity for gastrinoma and NEN of unknown primary however is much lower with 68% and 52% respectively [41]. As benign insulinomas are often small (0.5–2 cm) and have usually low expression of SSTR, correct localization is not always possible using ^68^Ga-SSTR-PET/CT [42]. Furthermore, since ^68^Ga-SSTR-PET/CTs show an inverse correlation with tumor grade and differentiation, ^18^F-Fluorodeoxyglucose (^18^FDG-PET/CT) is recommended for the evaluation of dedifferentiated and high-grade NENs [43] (details on ^18^FDG PET see Sect. 3.3 below). ^68^Ga-DOTATATE can also be utilized for the diagnosis and staging of lung NETs [32]. However, lower detection rates were described in atypical lung carcinoids, which often demonstrate a marked ^18^FDG-PET uptake [44]. Staging of patients with pheochromocytoma or paraganglioma using ^68^Ga-SSTR-PET shows a superiority for evaluation of metastatic disease compared to ^131^metaiodobenzygluanidine (MIBG)-scans and ^18^FDG-PET [45, 46]. Patients with medullary thyroid carcinoma show adequate SSTR receptors for imaging only in < 30% of cases (Reubi and Waser 2003). Therefore, diagnostic sensitivity with ^68^Ga-SSTR-PET/CT can be limited in those tumors.

While most NENs show a high affinity for ^68^Ga-SSTR-PET, it is important to keep in mind that other neoplasms like renal cell carcinoma, melanoma or meningioma, or systematic inflammatory diseases like sarcoidosis and lymphoma are also known to express SSTR which can lead to false-positive findings [47].

In summary, ^68^Ga-SSTR-PET/CT provides high diagnostic sensitivity for most NEN types and is recommended as the standard molecular imaging technique for patients with NEN.

#### SSTR Antagonist

While ^68^Ga-DOTATOC/-TATE and -NOC use somatostatin agonists, an alternative approach is the use of SSTR antagonists. SSTR agonists are characterized by internalization and intracellular retention following their binding to the SSTR and therefore depend on active receptors. SSTR antagonists, however, are not limited to the active receptors, leading to a several fold more binding sites and consequently increased tumor uptake [48]. This resulted in a higher tumor-to-background ratio, a longer tumor retention time, and higher tumor uptake for the SSTR antagonist in ex vivo autoradiography of human NEN samples [49]. These findings were confirmed with the developed PET-tracer ^68^Ga-OPS202 [50] which showed a higher tumor uptake than ^68^Ga-DOTATATE in murine studies. The superiority of the SSTR antagonist was confirmed clinically by Nicolas et al. [51•], showing a higher lesion-based overall sensitivity and higher detection rate of liver metastasis [52].

Taken together, while detection and staging of NENs with an SSTR-antagonist PET/CT is promising and might become the new standard, further clinical studies confirming the current data are needed.

#### Copper-SSTR Imaging

Another alternative to ^68^Ga-based SSTR imaging lies in the use of copper radioisotopes in combination with SSTR agonists or antagonists. To date, ^64^Cu seems to have the most potential to make its way into clinical practice. This is on the one hand due to its longer half-life of around 13 h which is convenient in clinical practice as it opens the scanning window to up to 3 h [53]. In addition, its ability to build stable complexes to different chelators and its potential for higher spatial resolution due to its decay mode is an advantage.

In 2012, the first in human study with ^64^Cu-DOTATATE involving 14 NEN patients and comparing the results to SPECT/CT was published [54]. The results indeed showed high image quality and spatial resolution, documenting additional lesions in 43% of patients. A later head-to-head comparison with ^111^In-DTPA-Octreotide in 112 NEN patients [55] confirmed the superior sensitivity and diagnostic accuracy of ^64^Cu over ^111^In (97% and 97% versus 87% and 88%, respectively). A direct comparison of ^64^Cu-DOTATATE with ^68^Ga-DOTATOC in 59 patients showed a sensitivity of 100% and specificity of 90% for both scans to diagnose NEN disease [56]. While in this study radiation dose was significantly higher for the ^64^Cu-DOTATATE compared to the ^68^Ga-DOTATOC (5.7–8.9 mSv vs 2.8–4.6 mSv), a recent dose-ranging study determined the optimal dose at 4.0 mSv to obtain high-quality diagnostic imaging [57].

Accordingly, ^64^Cu-SSTR-PET might become a diagnostic alternative for NEN centers without access to ^68^Ga.

### Alternatives to Somatostatin Receptor Imaging

#### Currently Available Imaging Techniques

##### 18F-FDG PET

^118^F-FDG is established as a diagnostic option for patients with negative lesions on SSTR-PET/CT. As tumors such as poorly differentiated grade 3 NENs have a high-glucose turnover rate, they can be visualized by ^18^F-FDG PET/CT [58]. Using a dosage of 4 MBq ^18^FDG per kg body weight, PET scan is performed 60 min after administration. ^18^FDG-PET has a high spatial resolution (4–6 mm) and can be hybridized with CT or MRI. Patient radiation dose for ^18^FDG is around 3.5 mSv [8•].

While ^18^F-FDG has a poor sensitivity for low-grade lesions due to the limited tracer uptake, it has diagnostic and prognostic value in higher-grade lesions [58–60]. An evaluation of 69 pancreatic NEN patients showed the clinical usefulness of ^18^F-FDG PET/CT in identifying progression of disease with unfavorable clinical outcome with a high diagnostic accuracy [61]. ^18^F-FDG PET also showed high uptake for SDHx-related pheochromocytoma or paraganglioma [62].

In the last years, the combined use of SSTR PET/CT and FDG PET/CT imaging has been proposed for the thorough evaluation of patients with NENs [28, 63]. Although this dual approach is complementary, it is currently rarely used due to financial and radiation constraints.

##### 18F-DOPA

Fluorine F-18 fluoro-dihydroxyphenylalanine (^18^fluorodopa or ^18^F-DOPA) is the amino acid analog fluorodopa (FDOPA) labelled with fluorine F18, a positron-emitting isotope. ^18^F-DOPA is taken up into the cells via the neutral amino acid transporter. ^18^F-DOPA is an alternative PET tracer for countries where ^68^Ga-SSTR imaging is not available, as it showed superior diagnostic sensitivity to SSTR-scintigraphy [64]. However, when compared to ^68^Ga-SSTR-PET, ^18^F-DOPA was inferior and comes at a higher radiation dose and cost [65, 66].

However, there is a place for ^18^F-DOPA imaging in SSTR-negative NENs, especially in medullary thyroid cancer (MTC) where it showed a predictive value [67]. In a study evaluating 60 patients with MTC 6 months after surgery, 27 showed abnormal findings while 33 scans remained unremarkable [68]. The patients with the unremarkable scans had a longer disease-specific survival rate.

Another possible role has been described in the diagnosis of nesidioblastosis in the differential diagnosis of endogenous hyperinsulinemia [69] as well as for the evaluation of pheochromocytoma and paraganglioma [70]. Overall, however, ^18^F-DOPA PET is more of a second line modality and ^68^Ga-SSTR-PET should be used first, if available.

##### GLP-1 Receptor Imaging

The glucagon-like peptide-1 receptor (GLP-1R) is another targetable peptide hormone receptor and is mainly localized on pancreatic beta cells [65]. This makes GLP-1R interesting for imaging of insulinomas, which are challenging to diagnose due to their small size and anatomical proximity to the kidneys. Furthermore, nesidioblastosis is a rare differential diagnosis of endogenous hyperinsulinemic hypoglycemia, which needs a different therapeutic approach [71]. Importantly, insulinoma usually express a low number of SSTR resulting in a low detection rate using SSTR imaging. However, GLP-1R are expressed with a high incidence and at high density in insulinomas [72].

**Fig. 2:**
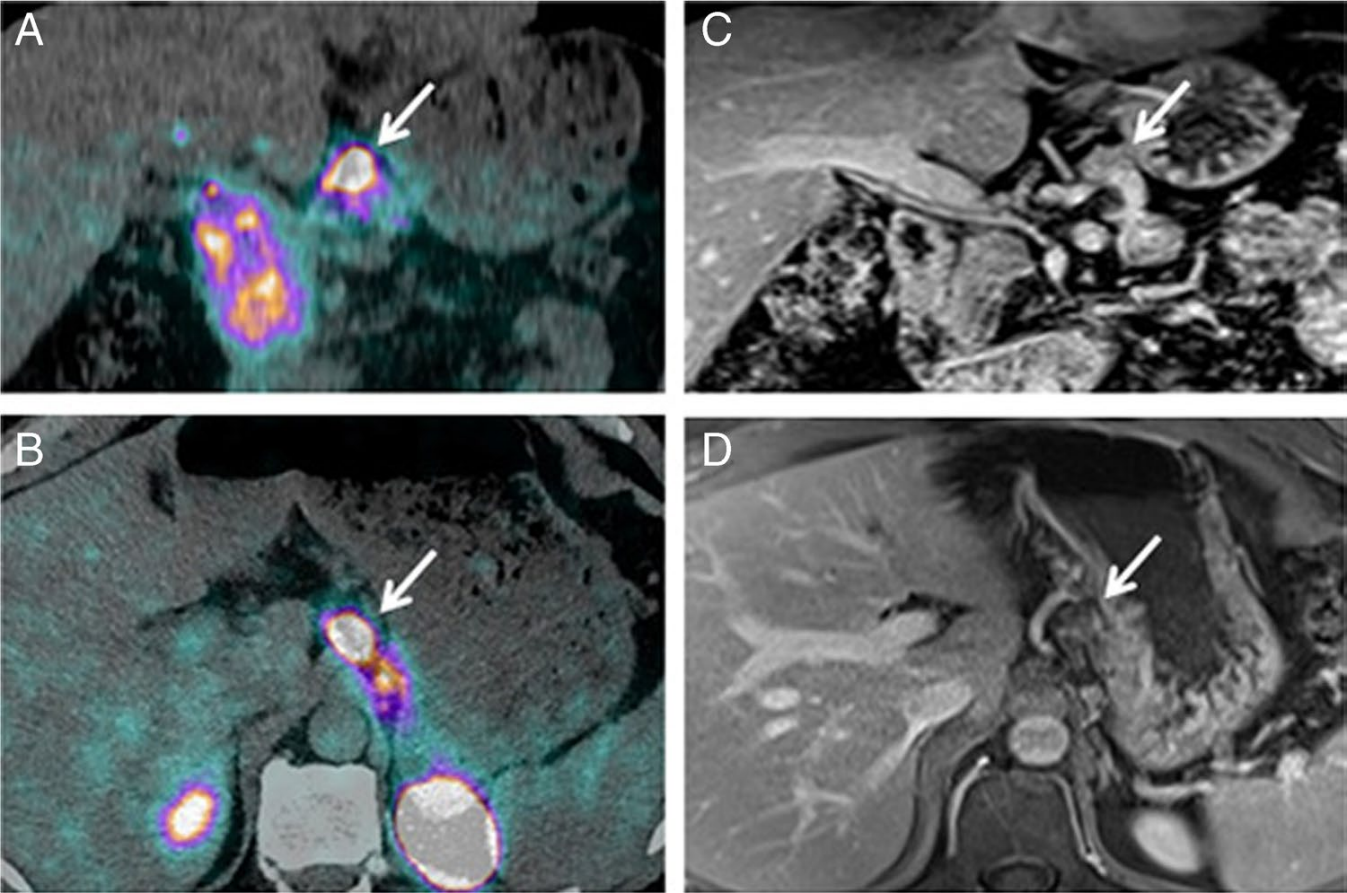
Targeting of GLP-1R with 68Ga-DOTA-exendin-4 PET/CT. Patient with biochemically confirmed endogenous hyperinsulinemic hypoglycemia. CT and MRI were negative. Coronal (**A**) and transaxial (**B**) PET/CT showed focal ^68^Ga-DOTA-exendin-4 uptake in the body of the pancreas (white arrows) consistent with a benign insulinoma. Coronal (**C**) and transaxial (**D**) T1-weighted MRI showed a slightly hypointense signal at the same location (white arrows) and was only retrospectively interpreted as a suspicious lesion. Histology confirmed an insulinoma in the pancreatic body

As the natural GLP-1 ligand is rapidly degraded by dipeptidyl-peptidase-4 (DPP4) [73], the DPP4-resistant GLP-1 analogue exendin-4 was developed. Using the radioisotope Indium-111, exendin-4 was then coupled via the chelator DTPA, leading to the radiotracer ^111^In-DTPA-exendin-4 [74, 75]. The initial promising proof-of-principle data [75, 76] were later confirmed in a multicentre study [77]. In this study of 30 insulinoma patients, ^111^In-DTPA-exendin-4-SPECT/CT showed superior diagnostic sensitivity to conventional MRI. The final break-through was achieved, when exendin-4 was coupled to ^68^Ga-DOTA allowing for PET imaging [78]. In a prospective study evaluating 52 patients with suspected benign insulinoma, ^68^Ga-DOTA-exendin-4 PET/CT showed a higher diagnostic accuracy of 94% compared to 68% with ^111^In-DOTA-exendin-4 SPECT/CT and standardized 3-Tesla-MRI (Fig. 2) [79•]. Furthermore, ^68^Ga-DOTA-exendin-4 PET/CT also proved useful in the evaluation of the usually multiple pancreatic lesions in patients with multiple endocrine neoplasms type 1 (MEN-1) patients [80].

With its additional advantage of having a shorter investigation time with a lower radiation burden, ^68^Ga-DOTA-exendin-4 PET/CT was proposed the diagnostic method of choice for suspected insulinoma negative on conventional imaging, thereby avoiding the cumbersome selective intra-arterial calcium stimulation and venous sampling test. As DOTA-exendin-4 can induce hypoglycemia, a concomitant glucose-infusion is recommended. Transient nausea and vomiting were also reported shortly after injection of the tracer.

Expression of GLP-1R is limited in malignant insulinoma; however, they often express SSTR making them eligible for imaging with ^68^Ga-SSTR-PET [81].

##### MIBG

Neuroendocrine cells express the norepinephrine transporter, allowing norepinephrine and its structurally similar guanethidine derivative metaiodobenzygluanidine (MIBG) to enter the cell. This transporter is consequently also expressed on several NENs, such as pheochromocytoma and paraganglioma, gastrointestinal NENs and MTC [82]. The iodine-123 labelled MIBG is used for diagnosis and staging in gastrointestinal NENs, pheochromocytoma/paraganglioma and MTC [82–84]. In direct comparison however, radiolabelled MIBG showed lower sensitivity compared to the ^68^Ga-based SSTR imaging [82].

#### Future Developments

##### FAP/FAP-Inhibitors

The fibroblast activation protein (FAP) is a serine proteinase which is overexpressed on the cell surface of activated fibroblasts, particularly cancer-associated fibroblasts in tumor stroma [12]. FAP-specific inhibitors have recently been developed as radioligands for PET imaging [85]. ^68^Ga-FAPI-04 has low nanomolar affinity to FAP, is almost completely internalized, has a rapid blood clearance, and showed excellent image contrast in across many tumor entities [86]. As small intestinal NENs are characterized by extensive fibrosis surrounding the primary tumor and mesenteric metastases, potentially leading to intestinal obstruction and ischemic complications, FAPI-based PET imaging could be particularly interesting in these patients.

##### CXCR4

The C-X-C motif chemokine receptor 4 (CXCR4) is another receptor which is overexpressed on dedifferentiated SSTR2 negative NENs [87, 88]*.* Coupled to ^68^Ga as the diagnostic compound ^68^Ga-Pentixafor, CXCR4 expression can be reliably assessed in vivo [89, 90].

In a direct comparison of ^68^Ga-Pentixafor with ^68^Ga-DOTATOC- and^18^F-FDG-PET/CT in 12 patients with GEP NENs, ^68^Ga-based SSTR imaging showed a clear diagnostic superiority of 92%, followed by 83% for ^18^F-FDG and 50% for ^68^Ga-Pentixafor [89]. However, being negative in all G1 patients, the diagnostic accuracy of ^68^Ga-Pentixafor increased with increasing tumor grade (50% G2, 80% G3 patients). But since all CXCR4-positive lesions also showed high ^18^F-FDG-uptake, there is currently no additional value of this imaging technique in NENs.

##### GIPR

Another member of the gut peptide family is the glucose-dependent insulinotropic polypeptide receptor (GIPR). GIP shows similar characteristics to GLP-1 including glucose-dependent insulin secretion and inactivation by DPP4 [73]. While in normal tissue, only low expression of GIPR is detected, they are overexpressed in insulinoma and gastrinoma, as well as non-functioning pancreatic, ileal, and lung NENs [91]. Also, for the around 10% of NENs which do not express SSTR nor GLP-1R, GIPR is an attractive target since it is expressed in the majority of those tumors [91]. While pre-clinical data showed promising results, the translation of those findings into clinical practice is currently pending.

##### CCK2R

Patients with MTC are often diagnosed with metastatic disease, and systemic therapeutic options are limited [92]. Already in 1997, the expression of the transmembrane G-protein coupled cholecystokinine-2 receptor (CCK2R) was described to be present on 90% of MTCs [93]. Since those tumors usually have a low incidence of SSTR expression [72], the CCK2R constitutes an attractive alternative target for peptide-based molecular imaging.

Minigastrin is a peptide CCK2R agonist, used for in vivo visualisation of its expression. In 2016, the detection of an MTC in one patient was reported using the ^68^Ga-labelled minigastrin analogue MG48 (^68^Ga-PP-F11) PET/CT [94]. Several CCK2R targeting peptides are being evaluated at the moment, promising improved diagnostic evaluation for future MTC patients [95•].

### Artificial Intelligence Tools

Radiomics, defined as the use of advanced computer analysis and deep learning techniques to find and quantify imaging, have the potential to lead to more differentiated grading, even allowing prediction of treatment response. In NENs, radiomics have so far mainly been evaluated in pancreatic NEN. Here, this technique was successfully used in several studies to differentiate low grade from high-grade tumors or pancreatic carcinomas [96, 97]. One study even showed a correlation between the developed radiomics nomogram and the proliferation markers Ki-67 and mitotic count [98]. Other studies have tried to use radiomics as a prognostic tool. However, while in one study the apparent diffusion coefficient (ADC) correlated with WHO tumor grade, no such association was found for recurrence-free survival [99]. Another study using ^68^Ga-DOTATOC PET/MRI showed decreased tumor lesion volume in responders but was also not able to predict treatment response to PRRT [100].

While these approaches are promising, their translation into clinical practice, especially prospective studies with patient relevant outcomes, are currently missing.

## Conclusion

Molecular imaging is a rapidly evolving field which will further improve current management of NENs. Among the currently available options, ^68^Ga-based SSTR PET/CT is recommended as the standard molecular imaging technique. Detection and staging of NENs with an SSTR-antagonist PET/CT is promising, but further clinical studies are needed. ^68^Ga-DOTA-exendin-4 PET/CT, using the GLP-1R-targeting peptide exendin-4, is likely to become the diagnostic method of choice for suspected insulinoma negative on conventional imaging.

For dedifferentiated NENs, new molecular targets such as the CXCR4 and the CCK2 receptors have been assessed, the latter showing intriguing data for the staging of MTC using the targeting ligand minigastrin.
